# Clinical Outcome in Patients With Intracerebral Hemorrhage Stratified by Type of Antithrombotic Therapy

**DOI:** 10.3389/fneur.2021.684476

**Published:** 2021-06-07

**Authors:** Merih Irem Baharoglu, Jonathan M. Coutinho, Henk A. Marquering, Charles B. Majoie, Yvo B. Roos

**Affiliations:** ^1^Department of Neurology, Amsterdam University Medical Centers—Location Academic Medical Center, Amsterdam, Netherlands; ^2^Department of Radiology and Nuclear Medicine, Amsterdam University Medical Centers—Location Academic Medical Center, Amsterdam, Netherlands; ^3^Biomedical Engineering and Physics, Amsterdam University Medical Centers—Location Academic Medical Center, Amsterdam, Netherlands

**Keywords:** antiplatelet therapy, antithrombotic therapy, intracerebral hemorrhage, oral anticoagulant, Vitamin K antagonist

## Abstract

**Background:** Antithrombotic therapy influences clinical outcome after spontaneous intracerebral hemorrhage (ICH). However, evidence on the effect of different antithrombotic therapies on outcome and a comparison between different therapies is scarce, while this is important for medical decision making. Therefore, we investigated clinical outcome after ICH stratified by type of antithrombotic therapy.

**Patients/Methods:** We performed a cohort study selecting consecutive ICH patients from our database, excluding patients without data on medication or therapeutic heparin use. Primary outcome was poor outcome (modified Rankin Scale ≥ 4) after 90 days. Secondary outcome was mortality at 90 days. We analyzed outcome and survival in patients with ICH using vitamin K antagonists (VKA), antiplatelet therapy (AP), and direct oral anticoagulant (DOAC) compared to no antithrombotic therapy adjusted for age, National Institutes of Health Stroke Scale (NIHSS), infratentorial localization, intraventricular extension, history of hypertension, diabetes, or stroke, and interaction between age and NIHSS.

**Results:** We included 916 patients (223 AP, 161 VKA, and 40 DOAC). VKA (adjusted odds ratio [aOR] 3.2, 95% confidence interval [CI], 1.6–6.3) and AP (aOR = 2.0, 95%CI: 1.1–3.7) were associated with poor outcome. DOAC use did not reach statistical significance (aOR = 2.4, 95%CI: 0.8–7.7). Patients who used any antithrombotic therapy had poorer survival compared to patients without antithrombotic treatment and patients using AP and DOAC had better survival compared to VKA after adjustment.

**Conclusions:** Patients with antithrombotic therapy have worse clinical outcome after ICH. Patients using VKA have higher risk of poor outcome and mortality compared to patients using AP. These findings highlight the deleterious effect of antithrombotic therapy in patients with ICH and stress the need for effective therapies for ICH patients.

## Introduction

Stroke is one of the leading causes of death and disability in the world. Although the majority of patients present with ischemic stroke, functional outcome and burden on society of patients with hemorrhagic stroke is worse (1). Outcome after spontaneous intracerebral hemorrhage (ICH) is highly variable and has not improved over the years (2). One of the most important factors that impacts outcome is hematoma volume and expansion (3). Antithrombotic treatment—in turn—is an important predictor of hematoma volume and expansion (4). For vitamin K antagonists (VKA), an association with poor outcome and mortality has been shown (5), however for antiplatelet therapy (AP) there are conflicting results in the literature weather its use is associated with poor outcome after ICH (6, 7). Moreover, most studies reporting on outcome after ICH report on short term mortality (8, 9) and have not compared ICH patients with different types of antithrombotic treatment (10). More data on clinical functioning of patients with ICH and comparison of patients with different antithrombotic therapies could help guide clinicians in their decision making for this patient group. We therefore investigated clinical functioning of patients at 90 days after ICH in relation to concomitant antithrombotic therapy.

## Methods

### Patients

We included consecutive adult (≥18 years) patients presenting with spontaneous ICH from January 2007 through September 2019 from a prospectively maintained registry in Amsterdam University Medical Centers—location Academic Medical Center. We excluded patients with traumatic ICH, proven underlying vascular malformations (aneurysm, arteriovenous- or cavernous malformation, and dural arteriovenous fistulas), hematological disease leading to coagulopathy, and hemorrhages in solid lesions. Patients without data on antithrombotic medication use and patients with heparin use (therapeutic or prophylactic) were also excluded. This study was evaluated by the medical ethical review board and the need for informed consent was waived. Information on patient demographics, medical history, co-morbidities, stroke severity on the National Institutes of Health Stroke Scale (NIHSS), Glasgow Coma Scale (GCS), antithrombotic therapy at time of ICH, blood pressure at presentation, characteristics of hemorrhage (intraventricular extension, supra-, or infratentorial localization), and medical therapy (i.e., reversal therapy, intensive care unit care, and operative intervention) was collected. The primary outcome was functional outcome scored with the modified Rankin scale (mRS) score (ranging from 0 through 6, where 0 represents patients without any symptoms and 6 represents patients who have died) at 90 days after stroke with use of a standardized interview either at the outpatient clinic or by telephone.

### Medical Management

Medical management of patients with ICH was based on standardized protocols in accordance with guidelines (11, 12). All patients were neurologically assessed and underwent head non-contrast CT imaging to confirm diagnosis. CT angiography or MRI was only performed when indicated by the treating physician. In all patients using VKA, the international standardized ratio (INR) was measured in the acute setting with a point of care device and VKA was antagonized as soon as possible with four factor prothrombotic complex concentrate (PCC, 25 IE per kg) in the emergency department, except for those in whom death was imminent on presentation. Reversal therapy for patients using a direct oral anticoagulant (DOAC) was either with four factor PCC (50 IE per kg) or a specific reversal agent (i.e., idarucizumab or andexanet-alpha). In patients using AP, reversal therapy with platelet transfusion was only given to patients included in a trial (13). If patients were comatose on admission, required airway stabilization, were surgically treated, or required intravenous anti-hypertensive medication, they were admitted to the intensive care unit (ICU). All others were admitted to a specialized stroke unit. Intravenous anti-hypertensive treatment was only possible in the ICU. Patients were transferred to the stroke unit after completion of intravenous treatment. Patient's consciousness was assessed every hour during the first 24 h of admission. For patients with a Glasgow coma scale (GCS) score of <13 with lobar ICH or hydrocephalus, a neurosurgical consult was obtained for either hematoma evacuation or ventricular drain placement.

### Statistical Analysis

Missing data was imputed for all analyses using multiple imputations. Patients were divided into four groups according to antithrombotic medication use at time of ICH: (1) none, (2) AP, (3) VKA, and (4) DOAC. Patients using both VKA and AP therapy were compared to patients using VKA only and these two groups were merged because baseline variables were comparable (Supplementary Table 1). The statistical program SPSS (IBM, version 21) was used. Statistical significance was set at *p* < 0.05. Student's *t*-test, Mann–Whitney *U*-test, and Chi-square test were used where appropriate. Odds ratio (OR) with 95% confidence intervals (CI) were calculated. The primary outcome was poor functional outcome defined as mRS 4 through 6. The association of antithrombotic treatments with poor functional outcome and mortality at 90 days was investigated using logistic regression and adjusted for known prognostic variables (age, NIHSS, infratentorial localization, intraventricular extension, and history of hypertension, diabetes, or stroke). We investigated whether there was an interaction between age and stroke severity on outcome (14) and also adjusted the analysis for this interaction if this was present. Sensitivity analyses additionally adjusting for ICU care was performed as well as separate analyses investigating in-hospital mortality and mortality at 30 days. We also compared outcome in patients using VKA only and those using both VKA and AP. A survival analysis of patients with different antithrombotic treatment was performed using Cox proportional hazards regression adjusting for age, NIHSS, history of stroke, intraventricular extension, infratentorial localization, and—if needed—interaction between age and NIHSS.

## Results

### Patients and Demographics

**Figure 1:**
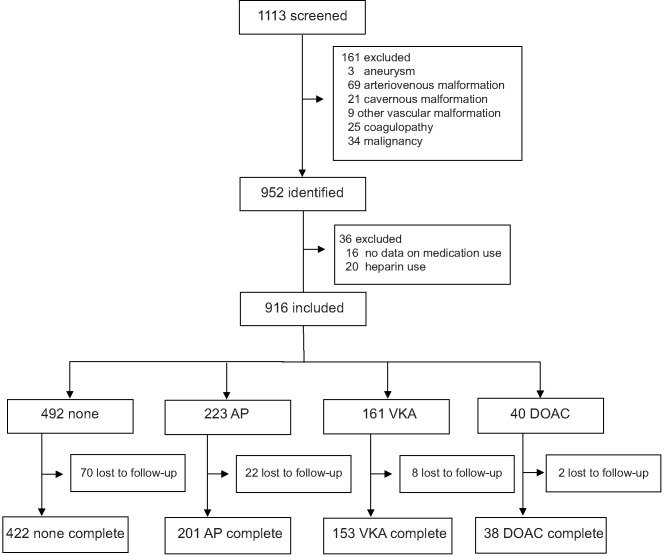
Flow diagram of included patients. AP, antiplatelet therapy; VKA, vitamin K antagonist; DOAC, direct oral anticoagulant.

We identified 952 patients with spontaneous ICH. Sixteen patients for whom medication use was not recorded and 20 patients who used heparin were excluded. In the remaining 916 patients, mean age was 67 years (range 20–98) and 484 (52.8%) were male. Four-hundred-ninety-two (53.7%) patients had no antithrombotic therapy, 223 (24.3%) used AP, 161 (17.6%) used VKA, and 40 (4.4%) used a DOAC at time of bleeding (Figure 1). On average, patients who used antithrombotic therapy were older and more often had a history of hypertension, diabetes, dyslipidemia, and stroke, compared to patients without antithrombotic treatment. Baseline stroke severity (NIHSS, GCS, intraventricular extension) was also worse in patients who used antithrombotic therapy (Table 1). When only comparing patients with different antithrombotic therapies (AP, VKA, or DOAC) there were no statistically significant differences in baseline characteristics, except that patients with AP more often had a history of diabetes or stroke.

**Table 1:** Baseline characteristics and medical management of patients with intracerebral hemorrhage without antithrombotic treatment (none), with antiplatelet therapy (AP), with vitamin K antagonists (VKA), and with direct oral anticoagulant (DOAC) use.

	**None (*n* = 492)**	**AP (*n* = 223)**	**VKA (*n* = 161)**	**DOAC (*n* = 40)**	***P*-value**
Age^*^	61.1 ± 15.6	72.7 ± 12.5	75.8 ± 11.2	78.4 ± 10.5	<0.001
Male^#^	260 (52.8%)	116 (52.0%)	90 (55.9%)	18 (45.0%)	0.648
Hypertension^#^	202 (41.0%)	132 (59.2%)	86 (53.4%)	24 (60.0%)	<0.001
Diabetes^#^	59 (12.0%)	57 (25.6%)	29 (18.0%)	6 (15.0%)	<0.001
Dyslipidemia^#^	27 (5.5%)	29 (13.0%)	23 (14.3%)	5 (12.5%)	0.001
Previous stroke^#^	43 (8.7%)	118 (52.9%)	40 (24.8%)	6 (15.0%)	<0.001
NIHSS^$^	14, 6–23	20, 9–28	17, 9–28	18, 6–34	<0.001
IVE^#^	160 (32.5%)	101 (45.3%)	61 (37.9%)	22 (55.0%)	0.001
MAP^$^	125, 109–143	121, 107–139	119, 103–135	124, 111–140	0.176
Infratentorial^#^	77 (15.7%)	29 (13.0%)	28 (17.3%)	10 (25.0%)	0.241
GCS^#^					0.001
3–4	56 (11.4%)	36 (16.1%)	32 (19.9%)	9 (22.5%)	
5–12	140 (28.5%)	87 (39.0%)	51 (31.7%)	10 (25.0%)	
13–15	296 (60.2%)	100 (44.8%)	78 (48.4%)	21 (52.5%)	
ICU care^#^	175 (35.6%)	56 (25.1%)	39 (24.2%)	13 (32.5%)	0.006
Ventricular drain^#^	58 (11.8%)	23 (10.3%)	7 (4.3%)	5 (12.5%)	0.046
Craniotomy^#^	56 (11.4%)	10 (4.5%)	5 (3.1%)	5 (12.5%)	0.001

*NIHSS, National Institutes of Health Stroke Scale; IVE, Intraventricular extension; MAP, Mean arterial pressure; GCS, Glasgow Coma Scale; ICU, intensive care unit*.

* *mean with standard deviation, ANOVA*;

# *totals with proportions, Chi-square test*;

$ *medians interquartile-range, Mann–Whitney U-test*.

### Medical and Surgical Treatment

Patients not using any antithrombotic therapy more often received ICU care and operative treatment (ventricular drain or craniotomy). There was no statistically significant difference in ICU care or craniotomy when comparing patients with different antithrombotic therapies. Patients with VKA were less likely to receive a ventricular drain (Table 1). All patients with VKA associated ICH had an INR > 1.5. One-hundred and ten patients (68.3%) with VKA received emergency reversal with four-factor PCC and 27 in the DOAC (67.5%) group. Additionally, one patient received idarucizumab and two andexanet alpha. Five patients with AP received platelet transfusion. Data on withdrawal of care was not available.

### Clinical Outcome and Mortality

**Figure 2:**
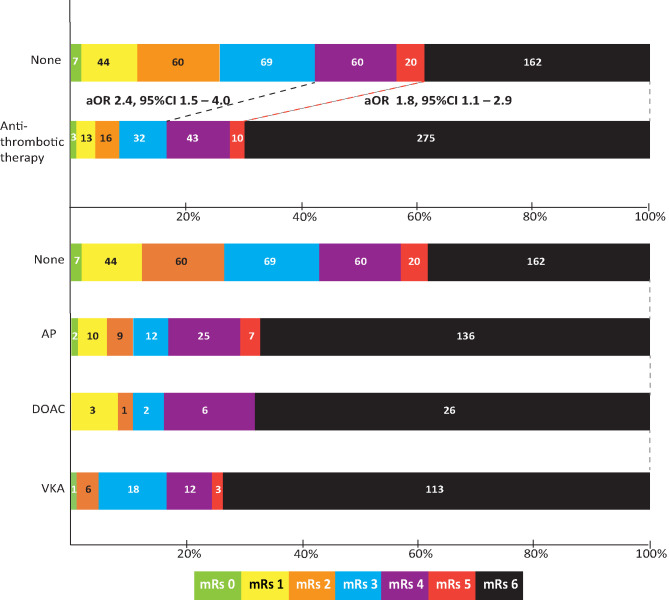
Outcome on the modified Rankin scale (mRs) of patients with intracerebral hemorrhage by type of concomitant antithrombotic therapy. Shown are scores for patients not using any antithrombotic therapy (none), using any type of antithrombotic therapy, and also split by type of concomitant antithrombotic therapy. Odds ratio is adjusted for age; history of hypertension, diabetes, or stroke; national institutes of health stroke scale; intraventricular extension; infratentorial localization, and interaction between age and NIHSS. aOR, adjusted odds ratio; CI, confidence interval; AP, antiplatelet therapy; VKA, vitamin K antagonist; DOAC, direct oral anticoagulant.

**Figure 3:**
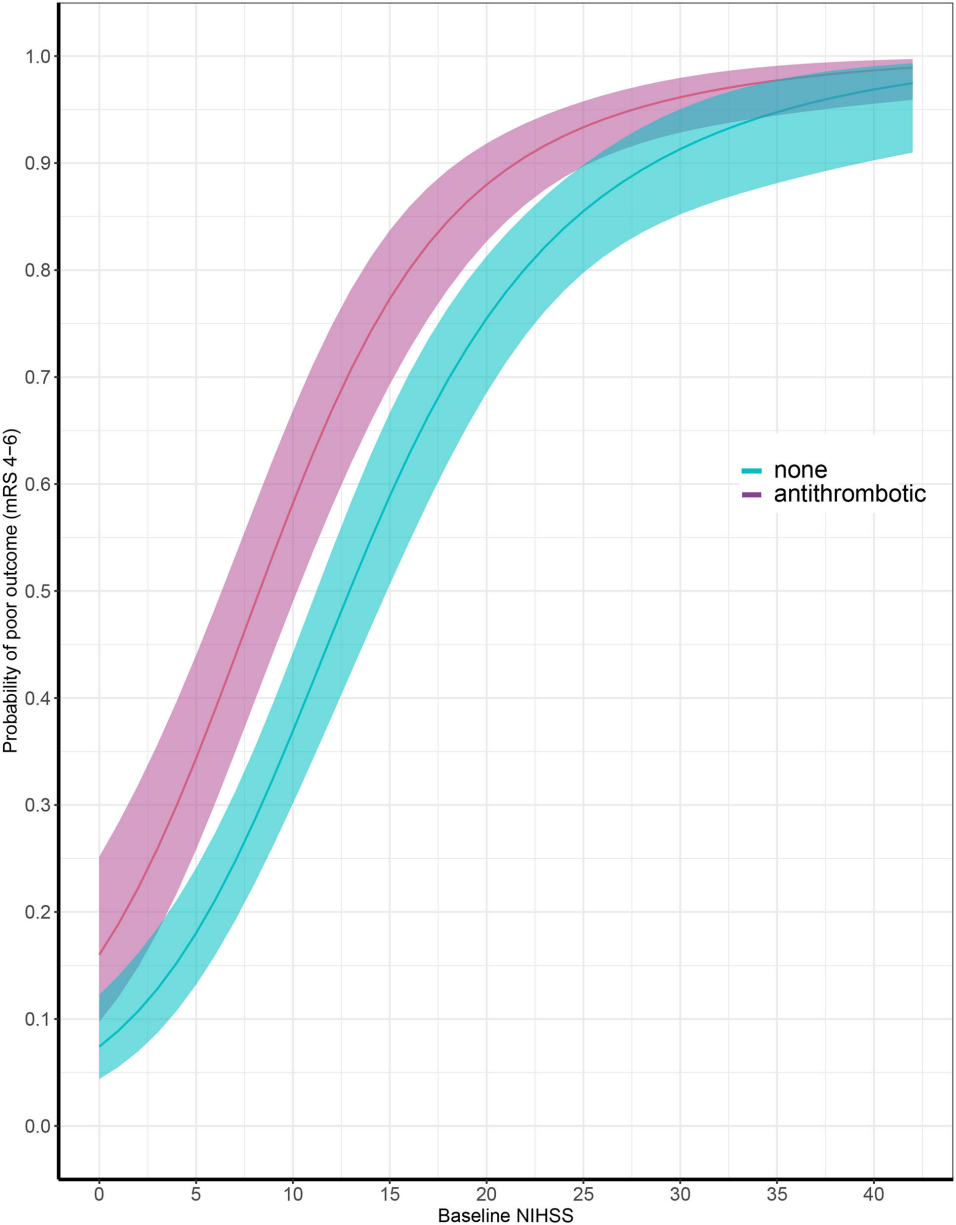
Probability of poor outcome according to baseline National Institutes of Health Stroke Scale score comparing patients with (in red) and without (in blue) antithrombotic treatment at time of ICH. *X*-axis showing baseline National Institutes of Health Stroke Scale (NIHSS) and *y*-axis showing probability of poor outcome at 90 days, defined as modified Rankin Scale (mRS) score of 4 through 6.

**Figure 4:**
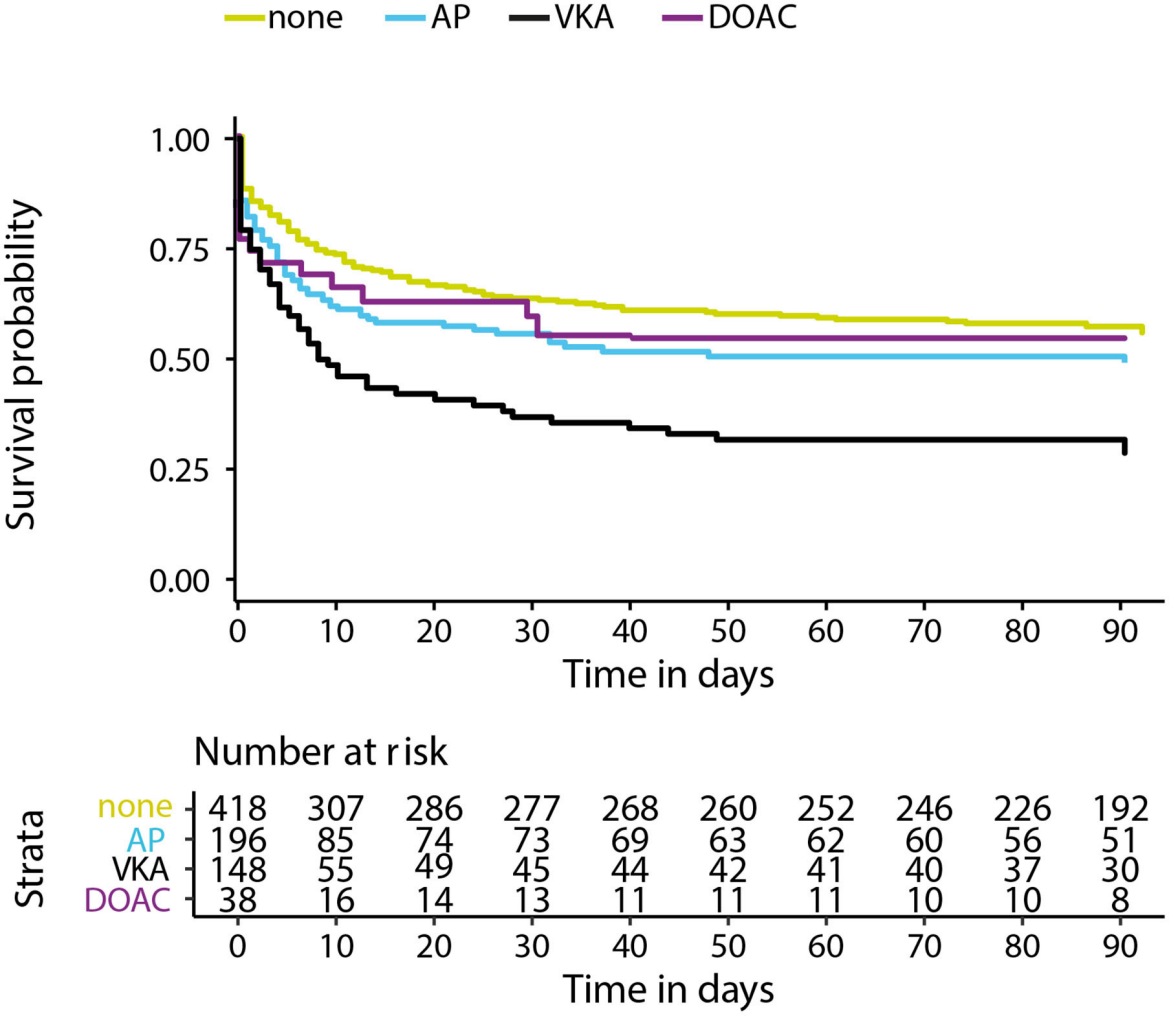
Kaplan–Meier survival curve in days for patients with intracerebral hemorrhage using no antithrombotic therapy (none), antiplatelet therapy (AP), vitamin K antagonists (VKA), or direct oral anticoagulants (DOAC). *X*-axis showing days from symptom onset and *y*-axis showing survival. Cox regression adjusting for age, NIHSS at baseline, history of stroke, intraventricular extension, infratentorial localization, and interaction for age and NIHSS showed a hazard ratio (HR) 1.4 and 95% confidence interval (CI), 1.0–1.8 for AP, HR = 1.8, 95%CI: 1.3–2.4 for VKA, and HR = 1.2, 95%CI: 0.8–1.9 for DOAC.

Data on functional outcome at 90 days was available for 814 (88.9%) patients and was imputed for the remainder. Median timing of outcome was 91 days (IQR 60–95). Patients with missing outcome were more likely not to use any antithrombotic treatment on admission and had less severe stroke symptoms (Supplementary Table 2). For an additional 14 patients (4 VKA, 5 AP, 5 none) the date of death was unknown excluding them from survival analysis. The overall mortality rate at 90 days was 53.7% (437 patients) and 70.0% (570 patients) had poor outcome. We found a significant interaction between age and NIHSS on poor outcome. Clinical outcome differed significantly between patients using any type of antithrombotic therapy and patients not using antithrombotic therapy [adjusted OR (aOR) for poor outcome 2.4, 95%CI: 1.5–4.0, and *p* < 0.0001, aOR for mortality 1.8, 95%CI: 1.1–2.9, *p* < 0.03; Figure 2]. Logistic regression analysis showed that AP (aOR = 2.0, 95%CI: 1.1–3.7) and VKA (aOR = 3.2, 95%CI: 1.6–6.3) use were independently associated with poor outcome. AP was not associated with mortality, while VKA use was. In patients using a DOAC, mortality and poor outcome rates were higher, but this difference did not reach statistical significance (Table 2). The probability of poor outcome according to baseline NIHSS was higher for patients who used any antithrombotic therapy compared to patients who did not use antithrombotic treatment (Figure 3). Sensitivity analysis adjusting for ICU care and on in-hospital and 30-day mortality showed similar results (Supplementary Tables 3, 4). A comparison of outcome of patients using VKA with and without AP was inconclusive due to the small number of patients using both treatments (Supplementary Table 5). Adjusted Cox regression showed that patients using AP (HR = 1.4, 95%CI: 1.0–1.8) and VKA (HR = 1.8, 95%CI: 1.3–2.4) had poorer survival compared to patients not using any antithrombotic therapy, with patients using VKA having worse survival compared to AP. DOAC use was not statistical significant (HR = 1.2, 95%CI: 0.8–1.9; Figure 4).

**Table 2:** Association of vitamin K antagonist (VKA), antiplatelet therapy (AP), and direct oral anticoagulant (DOAC) compared to no antithrombotic therapy use with poor functional outcome (Modified Rankin scale score ≥ 4) or mortality at 90 days.

		**Poor outcome**			**Mortality**	
	***N*, %**	**Unadjusted OR, 95%CI**	**Adjusted OR, 95%CI**	***N*, %**	**Unadjusted OR, 95%CI**	**Adjusted OR, 95%CI**
AP	168, 83.6	3.8, 2.5–5.8	2.0, 1.1–3.7	136, 67.7	3.3, 2.4–4.8	1.3, 0.7–2.2
VKA	128, 83.7	3.8, 2.4–6.1	3.2, 1.6–6.3	113, 73.9	4.6, 3.0–6.9	2.6, 1.3–5.3
DOAC	32, 84.2	4.0, 1.6–9.7	2.4, 0.8–7.7	26, 68.4	3.5, 1.7–7.2	1.5, 0.5–4.1

*Shown are numbers with proportions and odds ratio's (OR) with 95% confidence intervals (CI). Adjusted analyses were adjusted for age; history of hypertension, diabetes, or stroke; national institutes of health stroke scale (NIHSS); intraventricular extension (IVE); infratentorial localization; and interaction of age with NIHSS*.

## Discussion

Patients who used any type of antithrombotic therapy at time of ICH had worse outcome compared to patients who did not use antithrombotic therapy independent from age and baseline stroke severity. Mortality was higher in patients using VKA and did not reach statistical significance for patients using AP or DOAC. The mortality rate we found for all ICH patients was comparable to other studies (2, 15). Results on mortality and outcome for VKA associated ICH varies in the literature from around 30% in trials (16) to 50–77% in cohort studies (5, 17, 18). Mortality and morbidity rates reported for patients who used AP therapy varies even more in the literature. The largest cohort studies and meta-analyses report higher mortality rates in these patients (7), while other studies did not find an increased risk compared to patients without any antithrombotic treatment (6).

Outcome has not often been compared between different types of antithrombotic-therapy-associated ICH (19). Although we did not find an association between AP therapy and mortality, there was a clear association with poor outcome. This implies that many patients with AP-associated ICH do not die due to ICH, but do have severe morbidity. This finding is important because around a quarter of patients with ICH are using AP (20) and highlights the need for new therapy in these patients. While platelet transfusion in the acute setting seems like a plausible therapy, a recent trial failed to show a positive clinical effect and even suggested that platelet transfusion was, in fact, deleterious in these patients (13). More recent studies focusing on desmopressin, an agent thought to activate platelets, also didn't shown a positive effect on recovery (21, 22).

Poor outcome for VKA associated ICH was still 83% in our cohort, despite that two-third of patients received prompt reversal therapy. While a randomized trial showed that PCC is more effective than fresh frozen plasma in reversing effects of VKA on hemostasis and gives rapid normalization of INR (16), the effect of this therapy on clinical outcome seems to be at best marginal. With the introduction of DOAC's (23, 24) some have argued to prefer DOAC therapy over VKA, because of its lower risk of ICH (25). While some studies have found that patients with DOAC-associated ICH present with smaller hematomas, less severe stroke (26), and have better clinical recovery (27, 28), other studies found that patients with DOAC associated ICH have similar functional outcome and mortality compared to VKA-associated ICH (29). In our cohort the number of patients using DOAC was too small to draw conclusions. If patients with DOAC-associated ICH are indeed shown to have better outcomes, this could drastically change recommendations in treatment of patients requiring oral anticoagulation.

Our data also show that the poorer outcome in antithrombotic therapy associated ICH is—to some extend—independent of baseline stroke severity. When patients present with similar NIHSS scores, those who used antithrombotic therapy had a higher probability of poor outcome compared to patients who did not use antithrombotic treatment. The most plausible explanation for this finding is the higher risk of significant hematoma expansion after presentation in these patients. This also implies that there is a narrow window of opportunity to reverse antithrombotic therapy and improve clinical outcome. The lack of efficacy on clinical recovery of previous reversal trials could therefore be due to the timing of treatment, since most studies investigated treatment within 6–12 h of symptom onset. For effective reversal of antithrombotic therapy we likely need treatments that are given within the first hours of ICH onset to improve patient outcome. “Time is Brain” is most likely not limited to ischemic stroke, but could possibly be of even more importance in ICH.

In this study we used data from a consecutive and prospectively maintained cohort of patients with spontaneous ICH, thus reducing selection bias. All patients presenting with ICH were included in our database, even those that died shortly after presentation. Very few patients (1.4%) were excluded from this analysis due to missing data on medication use. Non-consecutive data from—for instance—a trial would have excluded those patients in whom death appeared imminent. This may explain why the mortality rate in our study is higher than that found in clinical trials (13, 16) and is more comparable to a recent cohort study (19). Our study however still has several limitations. One-hundred-two (11.1%) patients were lost to follow-up, and most of these patients were not using antithrombotic treatment. Although we imputed missing data for analyses, this could still have distorted some of our findings in outcome between patients with and without antithrombotic therapy use. Furthermore, since this is a single center cohort, generalizability could be limited, although patient treatment did abide to European (11) and United States standards (12). Difference in outcome according to concomitant antithrombotic therapy could be due to these patients receiving different medical treatment, especially since these patients were older. Therefor we performed a sensitivity analysis adjusting for ICU care, which did not change our results. Moreover we corrected for an interaction between age and stroke severity, known to effect medical decision making (14), but cannot exclude some residual confounding. Also data on timing of arrival to the hospital and door-to-needle time of reversal therapy for patients using VKA or DOAC was missing. Differences in timing could have affected some of the differences we found between the different antithrombotic therapy-associated ICH patients. Unfortunately, we were not able to measure hematoma volume at baseline, an important prognostic variable. Instead, we used the NIHSS and GCS to measure stroke severity. These clinical scores could be less reliable in ICH due to mass effect, edema, and intraventricular extension.

In summary our results further highlight the importance of antithrombotic therapy use at time of ICH onset on patient's prognosis. AP was associated with poor outcome, but not with mortality. This implies that many patients with AP-associated ICH have high morbidity. The association between antithrombotic therapy and clinical outcome is at least partially independent of baseline stroke severity. Unfortunately, treatment options are limited and effect of available reversal therapy on clinical outcome seems at best marginal. This could be partially due to timing of treatment. Our finding highlights the burden of antithrombotic therapy-associated ICH and the need for research on effective treatment and improved workflow.

## Data Availability Statement

The raw data supporting the conclusions of this article will be made available by the authors, without undue reservation.

## Ethics Statement

The studies involving human participants were reviewed and approved by Medisch Ethische Toetsingscommissie AMC. Written informed consent for participation was not required for this study in accordance with the national legislation and the institutional requirements.

## Author Contributions

Material preparation, data collection and analyses were performed by MB, under supervision of JC and YR. The first draft of the manuscript was written by MB and all authors commented on previous versions of the manuscript. All authors read and approved the final manuscript and contributed to the study conception and design.

## Conflict of Interest

HM reports to be cofounder and shareholder of Nico-lab. CM received funds from TWIN Foundation, CVON/Dutch Heart Foundation, Stryker, European Commission, Health Evaluation Netherlands (unrelated to this project; all paid to institution). CM and YB are shareholder of Nico-lab. The remaining authors declare that the research was conducted in the absence of any commercial or financial relationships that could be construed as a potential conflict of interest.
