# Supplementary material for: Clinical Outcome in Patients With Intracerebral Hemorrhage Stratified by Type of Antithrombotic Therapy
Source: Front Neurol. 2021 Jun 7;12:684476. doi: 10.3389/fneur.2021.684476 (PMC8215162; doi:10.3389/fneur.2021.684476)
Supplement: Supplementary file 1 [file Table_1.DOCX]

***Supplemental material***

**Article title: Clinical outcome in patients with intracerebral hemorrhage stratified by type of antithrombotic therapy**

Authors: M. Irem Baharoglu MD; Jonathan M. Coutinho MD PhD; Henk A. Marquering PhD; Charles B. Majoie MD PhD; Yvo B. Roos MD PhD

**Supplemental tables**

**Supplemental table 1: Analysis comparing baseline variable between patients using vitamin K antagonists (VKA) alone and patients using both VKA and antiplatelet therapy (AP).**

*Means with standard deviation, #numbers with proportions, ¥median with interquartile range

IVE = intraventricular extension, NIHSS = national institutes of health stroke scale, AP = antiplatelet therapy, VKA = vitamin K antagonist, DOAC = direct oral anticoagulant, NS=not significant

|  | **VKA (138)** | **VKA and AP (23)** | **significance** |
| --- | --- | --- | --- |
| Age* | 75.8 +/- 11.3 | 75.3 +/- 11.0 | NS |
| Male gender^#^ | 76 (55.1) | 13 (56.5) | NS |
| Hypertension^#^ | 73 (52.9) | 13 (56.5) | NS |
| Dyslipidemia^#^ | 19 (13.8) | 4 (17.4) | NS |
| Diabetes^#^ | 26 (18.8) | 3 (13.0) | NS |
| Previous stroke^#^ | 24 (23.5) | 6 (26.0) | NS |
| IVE^#^ | 55 (39.9) | 6 (26.0) | NS |
| Infratentorial^#^ | 24 (17.4) | 4 (17.4) | NS |
| NIHSS^¥^ | 18 (9 – 30) | 13.5 (3.5 – 18) | NS |
| GCS^#^  3-4  5-12  13-15 | 29 (21.0)  43 (31.2)  66 (47.8) | 3 (13.0)  8 (34.8)  12 (52.2) | NS |

**Supplemental table 2:** **Analysis comparing baseline variables between patient with (complete) and without (missing) outcome data.**

*Means with standard deviation, #numbers with proportions, ¥median with interquartile range

IVE = intraventricular extension, NIHSS = national institutes of health stroke scale, AP = antiplatelet therapy, VKA = vitamin K antagonist, DOAC = direct oral anticoagulant

|  | **Missing (102)** | **Complete (814)** | **significance** |
| --- | --- | --- | --- |
| Age* | 64.7 (16.5) | 67.6 (15.4) | 0.08 |
| Male gender# | 51 (50.0) | 433 (53.2) | 0.54 |
| Hypertension# | 43 (42.2) | 401 (49.3) | 0.26 |
| Dyslipidemie# | 10 (9.9) | 74 (9.1) | 0.65 |
| Diabetes# | 19 (18.6) | 132 (16.2) | 0.43 |
| Previous stroke# | 24 (23.5) | 183 (22.5) | 0.85 |
| IVE# | 31 (30.4) | 313 (38.5) | 0.11 |
| Infratentorial# | 12 (11.8) | 132 (16.2) | 0.26 |
| NIHSS¥ | 9.5 (4 – 15) | 17.5 (8 – 28) | **<0.001** |
| Antithrombotic therapy#  AP  VKA  DOAC | 22 (21.5)  8 (7.8)  2 (2.0) | 201 (24.7)  153 (18.8)  38 (4.7) | **0.005** |

**Supplemental table 3:** **Association of antiplatelet therapy (AP), vitamin K antagonist (VKA), and direct oral anticoagulant (DOAC) use with poor outcome and mortality at 90 days. Adjusted analysis using logistic regression with additional adjusting for ICU care.**

Shown are numbers with proportions and odds ratio´s (OR) with 95% confidence intervals (CI). Adjusted analyses were adjusted for age, history of hypertension, diabetes, or stroke, national institutes of health stroke scale (NIHSS), intraventricular extension (IVE), infratentorial localization, interaction of age with NIHSS, and ICU care.

|  |  | Poor outcome |  |  | Mortality |  |
| --- | --- | --- | --- | --- | --- | --- |
|  | *N, %* | *Unadjusted*  *OR, 95%CI* | *Adjusted*  *OR, 95%CI* | *N, %* | *Unadjusted*  *OR, 95%CI* | *Adjusted*  *OR, 95%CI* |
| AP | 168, 83.6 | 3.8, 2.5 – 5.8 | 2.0, 1.0 – 3.8 | 136, 67.7 | 3.3, 2.4 – 4.8 | 1.2, 0.7 – 2.2 |
| VKA | 128, 83.7 | 3.8, 2.4 – 6.1 | 3.1, 1.6 – 6.3 | 113, 73.9 | 4.6, 3.0 – 6.9 | 2.6, 1.3 – 5.3 |
| DOAC | 32, 84.2 | 4.0, 1.6 – 9.7 | 2.1, 0.7 – 6.8 | 26, 68.4 | 3.5, 1.7 – 7.2 | 1.4, 0.5 – 3.7 |

**Supplemental table 4:** **Association of antiplatelet therapy (AP), vitamin K antagonist (VKA), and direct oral anticoagulant (DOAC) use with mortality at 30 days and at discharge. Adjusted analysis using logistic regression.**

Shown are numbers with proportions and odds ratio´s (OR) with 95% confidence intervals (CI). Adjusted analyses were adjusted for age, history of hypertension, diabetes, or stroke, national institutes of health stroke scale (NIHSS), intraventricular extension (IVE), infratentorial localization, and interaction of age with NIHSS.

|  | Mortality discharge | | |  | Mortality 30days |  |
| --- | --- | --- | --- | --- | --- | --- |
|  | *N, %* | *Unadjusted*  *OR, 95%CI* | *Adjusted*  *OR, 95%CI* | *N, %* | *Unadjusted*  *OR, 95%CI* | *Adjusted*  *OR, 95%CI* |
| AP | 112, 55.7 | 2.8, 2.0 – 3.9 | 1.3, 0.8 – 2.2 | 121, 60.1 | 3.0, 2.2 – 4.2 | 1.4, 0.8 – 2.4 |
| VKA | 96, 62.7 | 4.1, 2.8 – 5.9 | 3.1, 1.8 – 5.3 | 105, 68.6 | 4.8, 3.3 – 7.9 | 3.5, 2.0 – 6.2 |
| DOAC | 24, 63.1 | 4.1, 2.1 – 8.0 | 2.3, 0.9 – 5.7 | 25, 65.8 | 4.5, 2.3 – 8.9 | 2.1, 0.8 – 5.4 |

**Supplemental table 5: Association of vitamin K antagonist (VKA) use with and without concomitant antiplatelet therapy (AP) use with poor outcome and mortality at 90 days. Adjusted analysis using logistic regression.**

Shown are numbers with proportions and odds ratio´s (OR) with 95% confidence intervals (CI). Adjusted analyses were adjusted for age, history of hypertension, diabetes, or stroke, national institutes of health stroke scale (NIHSS), intraventricular extension (IVE), and infratentorial localization.

|  | Poor outcome | | |  | Mortality 30days |  |
| --- | --- | --- | --- | --- | --- | --- |
|  | *N, %* | *Unadjusted*  *OR, 95%CI* | *Adjusted*  *OR, 95%CI* | *N, %* | *Unadjusted*  *OR, 95%CI* | *Adjusted*  *OR, 95%CI* |
| VKA w/o AP | 111, 80.4 | 2.9, 1.7 – 4.8 | 3.5, 1.7 – 7.4 | 97, 70.3 | 2.9, 2.0 – 4.4 | 2.4, 1.1 – 5.1 |
| VKA with AP | 17, 73.9 | 1.2, 0.5 – 3.1 | 1.2, 0.3 – 4.1 | 16, 69.6 | 2.1, 0.9 – 5.3 | 2.0, 0.7 – 6.2 |
